# Targeted mutagenesis using CRISPR/Cas system in medaka

**DOI:** 10.1242/bio.20148177

**Published:** 2014-04-11

**Authors:** Satoshi Ansai, Masato Kinoshita

**Affiliations:** Division of Applied Biosciences, Graduate School of Agriculture, Kyoto University, Kitashirakawa-Oiwake-cho, Sakyo-ku, Kyoto 606-8502, Japan

**Keywords:** Medaka, CRISPR/Cas, Genome editing, Mutagenesis, Off-target alterations

## Abstract

Clustered regularly interspaced short palindromic repeats (CRISPR)/CRISPR-associated (Cas) system-based RNA-guided endonuclease (RGEN) has recently emerged as a simple and efficient tool for targeted genome editing. In this study, we showed successful targeted mutagenesis using RGENs in medaka, *Oryzias latipes*. Somatic and heritable mutations were induced with high efficiency at the targeted genomic sequence on the *DJ-1* gene in embryos that had been injected with the single guide RNA (sgRNA) transcribed by a T7 promoter and capped RNA encoding a Cas9 nuclease. The sgRNAs that were designed for the target genomic sequences without the 5′ end of GG required by the T7 promoter induced the targeted mutations. This suggests that the RGEN can target any sequence adjacent to an NGG protospacer adjacent motif (PAM) sequence, which occurs once every 8 bp. The off-target alterations at 2 genomic loci harboring double mismatches in the 18-bp targeting sequences were induced in the RGEN-injected embryos. However, we also found that the off-target effects could be reduced by lower dosages of sgRNA. Taken together, our results suggest that CRISPR/Cas-mediated RGENs may be an efficient and flexible tool for genome editing in medaka.

## INTRODUCTION

Genome editing with artificial nucleases such as zinc-finger nucleases (ZFNs) and transcription activator-like effector nucleases (TALENs) has become a powerful tool for approaches involving reverse genetics in a wide range of organisms (Carroll, 2011; Joung and Sander, 2013). These enzymes efficiently induce site-specific DNA double-strand breaks (DSBs), resulting in targeted gene disruptions by insertions and deletions (indels) or targeted gene integrations by homologous recombination. However, since the DNA-binding domain of these nucleases determines their site specificity, re-engineering the binding domain sequence is essential for each new target site.

The type II clustered regularly interspaced short palindromic repeats (CRISPR)/CRISPR-associated (Cas) system has recently emerged as an RNA-guided endonuclease (RGEN) for targeted genome editing. CRISPR and Cas proteins are essential components of the adaptive immune system in bacteria and archaea to detect and silence invading viruses and plasmids (Wiedenheft et al., 2012). In type II CRISPR system, Cas9 protein, CRISPR RNAs (crRNAs), and trans-activating crRNA (tracrRNA) form ribonucleoprotein complexes that induce site-specific DNA cleavage guided by crRNAs (Gasiunas et al., 2012; Jinek et al., 2012). The recognition specificity of Cas9 endonuclease from *Streptococcus pyogenes* type II CRISPR/Cas system can be programmed only by a synthetic single-guide RNA (sgRNA) consisting of a fusion of crRNA and tracrRNA (Jinek et al., 2012). Recent studies have shown that Cas9 and engineered sgRNA are the only components necessary and sufficient for targeted DNA cleavage and efficient genome editing in cultured human cells (Cho et al., 2013; Cong et al., 2013; Fu et al., 2013; Hsu et al., 2013; Mali et al., 2013), mice (Wang et al., 2013), *Drosophila* (Bassett et al., 2013; Gratz et al., 2013), *Caenorhabditis elegans* (Friedland et al., 2013), and zebrafish (Chang et al., 2013; Hwang et al., 2013a; Hwang et al., 2013b; Jao et al., 2013; Xiao et al., 2013). Because of its simple customizing process compared to the assembling of TALEN or ZFN modules, the CRISPR/Cas-mediated RGENs have the potential for being developed as a robust and efficient tool for genome editing.

However, RGENs still pose several unanswered questions for research applications (Mussolino and Cathomen, 2013). It remains unclear whether the selection of the target sequence is crucial to achieve effective targeted cleavage. Site specificity of DNA cleavage by the Cas9 endonuclease from *S. pyogenes* depends on two factors, one being the base-pair complementarity between the first 20 nucleotides (nts) of a guide RNA and a target DNA sequence, and the other being the sequence “NGG”, referred to as the protospacer adjacent motif (PAM), adjacent to the complementary region in the target site (Jinek et al., 2012). Hwang et al. described that sgRNAs transcribed by a T7 RNA polymerase require their target sequence in the form 5′-GG-N_18_-NGG-3′ because GG is added at the 5′ ends of the transcripts initiated at the T7 promoter (Hwang et al., 2013b). On the other hand, a study in cultured human cells showed that double mismatches at the 5′ ends of the sgRNAs are tolerated (Fu et al., 2013), and in vitro studies showed that the GG required by the T7 promoter do not affect cleaving activities (Cho et al., 2013; Jinek et al., 2012). Furthermore, Ran et al. showed that 5′ extension of sgRNA sequences could not contribute to Cas9 targeting specificity in cultured human cells because the 5′ extensions were processed (Ran et al., 2013a). In fact, Hwang et al. also reported that sgRNAs transcribed by T7 polymerase could target the genomic sequences without the 5′ ends GG in zebrafish (Hwang et al., 2013a). However, there are very few in vivo studies that investigate the effects of the 5′-end sequences on the cleaving activities. Second, and more importantly, the relatively short target sequence of 20 bp or fewer for the RGENs raises questions about their specificity. It was reported that potential off-target sites including both a PAM and a perfect match of at least 12 bp at the 3′ end of the 20-bp targeting sequence were not disrupted in mice (Wang et al., 2013), *Drosophila* (Bassett et al., 2013; Gratz et al., 2013), and *C. elegans* (Friedland et al., 2013). However, off-target sites harboring up to five mismatches were mutagenized in cultured human cells (Cho et al., 2014; Fu et al., 2013; Hsu et al., 2013) and zebrafish (Jao et al., 2013). Additionally, a more recent work indicates that the off-target sites that have up to 3 bp mismatches except in the 8-bp sequence adjacent to a PAM can be disrupted by RGENs (Wang et al., 2014). These suggest that the RGENs can also induce off-target alterations in other organisms.

Here, we showed successful targeted mutagenesis using CRISPR/Cas-mediated RGENs in medaka (*Oryzias latipes*), which is a small freshwater teleost and is used as a vertebrate model in a wide range of scientific studies (Takeda and Shimada, 2010; Wittbrodt et al., 2002). We injected fertilized eggs with capped RNAs encoding the *S. pyogenes* Cas9 nuclease and sgRNAs transcribed by the T7 promoter and introduced somatic and germ line mutations in their target sites with frequencies comparable to TALEN-mediated mutagenesis studied previously by us (Ansai et al., 2013). We showed that the RGENs introduced mutations at the target genomic sequence without GG at the 5′ end as efficiently as those starting with GG. We also revealed that the off-target alterations in the RGEN-injected fish were introduced at genomic loci harboring double mismatches in the 18-bp sequence located at the 3′ ends of the targeting sequence. Additionally, we exhibited that these off-target effects were reduced by lower dosages of sgRNA.

## MATERIALS AND METHODS

### Fish

A d-rR strain was used in this study. Fish were maintained in an aquarium with recirculating water in 14/10-h day/night cycle at 26°C. The care and use of animals in this study were in accordance with the guidelines of the Animal Experimentation Committee of Kyoto University.

### Cas9 nuclease expression vector

A Cas9 expression vector for SP6 in vitro transcription, pCS2+hSpCas9, was generated in this study. DNA sequence encoding the human codon-optimized *S. pyogenes* Cas9 nuclease was amplified from the pX330 (Addgene Plasmid 42230) (Cong et al., 2013) by PCR using the primers: hSpCas9FW and hSpCas9RV (supplementary material Table S1). The resulting PCR product was cloned into the BamHI/XbaI site of pCS2+MT vector (Turner and Weintraub, 1994). This expression vector will be made available via Addgene (http://www.addgene.org).

### sgRNA expression vector

The pDR274 vector (Addgene Plasmid 42250), harboring a T7 promoter positioned upstream of a partial guide RNA sequence (Hwang et al., 2013b) was used for sgRNA expression. Appropriately designed oligonucleotides were synthesized with oligonucleotide purification cartridge (OPC) purification at Operon Biotechnologies (Tokyo. Japan). A pair of oligonucleotides (final concentration: 10 µM each) was annealed in 10 µL of annealing buffer (40 mM Tris-HCl [pH 8.0], 20 mM MgCl_2_, and 50 mM NaCl) by heating to 95°C for 2 min and then cooling the mixture slowly to 25°C in 1 h. The pDR274 vector was digested with BsaI-HF (New England Biolabs), and the annealed oligonucleotides were ligated into the pDR274 vector. Sequences of the genomic target sites and the annealed oligonucleotides are listed in supplementary material Table S2.

### RNA synthesis and microinjection

The Cas9 expression vector was linearized by NotI digestion. Capped RNA was synthesized using the mMessage mMachine SP6 Kit (Life Technologies), and then purified using the RNeasy Mini Kit (Qiagen). The sgRNA expression vectors were digested by DraI, and the sgRNAs were synthesized using the AmpliScribe T7-Flash Transcription Kit (Epicentre). The synthesized sgRNAs were purified by ammonium acetate precipitation.

These RNA sequences were diluted to appropriate concentrations and injected approximately 2–4 nL of the RNA mixture into fertilized eggs before the first cleavage, as described previously (Kinoshita et al., 2000).

### Genomic DNA extraction

Embryos were lysed individually at 3 days post fertilization (dpf) in 25 µL of alkaline lysis buffer containing 25 mM NaOH and 0.2 mM EDTA (pH 8.0) and incubated at 95°C for 15 min after breaking the egg envelope (chorion) with forceps. Samples were neutralized with 25 µL of 40 mM Tris-HCl (pH 8.0) and used as genomic DNA samples.

### Heteroduplex mobility assay

Heteroduplex mobility assay (HMA) was performed to detect RGEN-induced mutations (Ansai et al., 2014; Chen et al., 2012; Ota et al., 2013). A 146-bp fragment containing the entire genomic target sequence of the *DJ-1* gene was amplified using primers DJ1-FW2 and DJ1-RV2 (supplementary material Table S1). The reaction mixture contained 1 µL of genomic DNA as template, 1× PCR buffer for KOD FX, 0.4 mM of each dNTP, 0.2 µM of each primer, and 0.05 unit of KOD FX (Toyobo) in a total volume of 10 µL. The cycling conditions were as follows: one cycle at 94°C for 2 min, followed by 35 cycles of 98°C for 10 sec, 56°C for 20 sec, and 68°C for 20 sec. The resulting amplicons were analyzed using a microchip electrophoresis system (MCE-202 MultiNA; Shimazu) with the DNA-500 reagent kit.

### Sequence analysis for somatic mutations

For sequence analysis at *DJ-1* locus, the genomic region including the target site of sgRNAs was amplified with KOD -plus- Neo DNA polymerase (TOYOBO) using the primers DJ1-FW3 and DJ1-RV3 (supplementary material Table S1). The PCR conditions were as follows: one cycle at 94°C for 2 min, followed by 35 cycles of 98°C for 10 sec, 58°C for 30 sec, and 68°C for 30 sec. The PCR amplicons were subcloned into the EcoRI/XhoI site of the pBluescript KS II (+) vector. The fragment containing the cloned genomic sequence was amplified from each colony using the M13 forward and reverse primers (supplementary material Table S1). Each fragment was sequenced using a T7 promoter primer (supplementary material Table S1).

### Quantification of mutations with restriction fragment length pattern (RFLP)

A 285-bp fragment including the genomic target sequence of the sgRNA was amplified using the primers DJ1-FW2 and DJ1-RV1 (supplementary material Table S1). The reaction mixture contained 2 µL of genomic DNA template, 1× reaction buffer, 0.8 mM of each dNTP, 1.5 mM of MgCl_2_, 0.2 mM of each primer, and 0.5 unit of HybriPol DNA Polymerase (Bioline, London) in a total volume of 20 µL. The cycling conditions were as follows: one cycle at 95°C for 2 min, followed by 30 cycles of 95°C for 20 sec, 58°C for 30 sec, and 72°C for 30 sec. The resulting product was precipitated with ethanol for buffer exchange and was digested at 37°C for overnight in 10 µL of the solution containing 1× L buffer and 2 units of the AluI restriction enzyme. After inactivation at 80°C for 10 min, the digested fragments were analyzed using a microchip electrophoresis system (MCE-202 MultiNA; Shimazu) with the DNA-500 reagent kit. The molar concentrations of both digested and undigested fragments were quantified using the MultiNA Viewer software. The mutation rate was calculated as the ratio of the undigested fragment to the sum of the undigested fragment and the larger digested fragment as described previously (Ansai et al., 2013).

### Off-target analysis

Potential off-target sites in the medaka genome were searched using a “Pattern Match” tool in New Medaka Map (beta) at the NBRP medaka web site (http://viewer.shigen.info/medakavw/patternmatch) with 2 criteria: criterion (i) perfect matching in the 12-bp sequence at the 3′ end of the 20-bp target sequence and the NGG PAM sequence; criterion (ii) matching of 16 to 18 bp of the 18 bp sequence at the 3′ end of the target followed by either the NGG or the NAG PAM sequence. All identified potential off-target sites were analyzed by HMA using the primers listed in supplementary material Tables S3 and S4, as described above. Subsequently, the genomic region containing altered off-target sites was amplified with TaKaRa Ex Taq (Takara) and subcloned into either the pGEM-T vector (Promega) or the T-Vector pMD20 (Takara). The fragment containing the cloned genomic sequence was amplified from each colony with the M13 forward and reverse primers (supplementary material Table S1), and then each fragment was sequenced using a T7 promoter primer or a SP6 promoter primer (supplementary material Table S1).

### Founder screening

RGEN-injected fish were mated with wild-type fish of the d-rR strain and genomic DNA was extracted from each F1 embryo. Mutation in each embryo was analyzed by HMA using the primers DJ1-FW2 and DJ1-RV2 (supplementary material Table S1). Mutant alleles in each embryo were determined by direct sequencing of the *DJ-1* gene region, amplified using the primers DJ1-FW2 and DJ1-RV1 (supplementary material Table S1).

### Statistical analysis

Mutation rates were analyzed with one-way ANOVAs followed by Tukey's HSD using the R language (http://www.r-project.org).

## RESULTS

### Introduction of somatic mutation at the medaka *DJ-1* locus

**Fig. 1.**
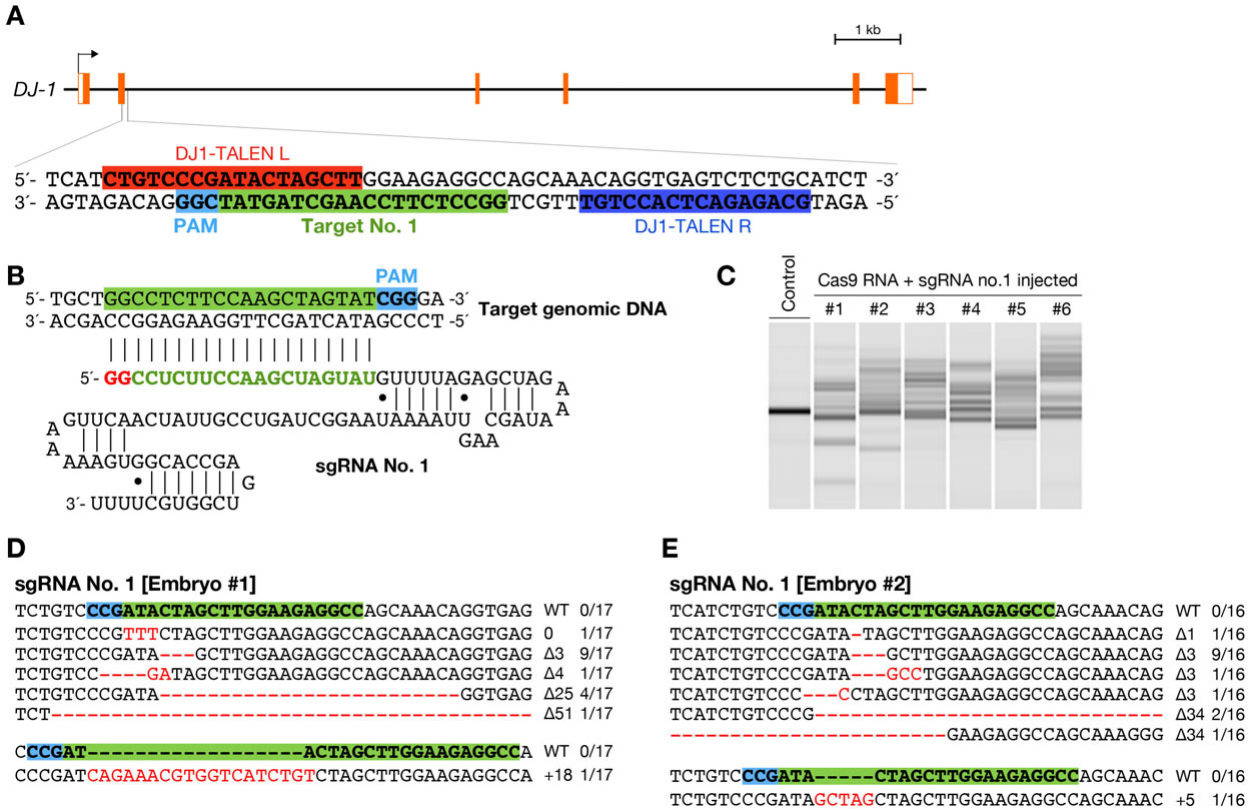
Induction of somatic mutations with CRISPR/Cas-mediated RGENs. (A) Schematic representation of the genomic structure of the *DJ-1* gene. Coding and untranslated exon regions are shown as solid and open boxes, respectively. The 20-bp target sequence of sgRNA no. 1 is indicated in green box, adjacent to NGG protospacer adjacent motif (PAM) sequence in light blue box. Red and blue boxes indicate the left and right recognition sequence of previously described TALENs (Ansai et al., 2013). (B) The sgRNA sequence for target site no. 1. First 20-nts sequence interacts with the complementary strand of the DNA target site. Red and green letters indicate the sequence required by T7 RNA polymerase and the customizable targeting sequence, respectively. (C) Heteroduplex mobility assay (HMA) in embryos injected with a mixture containing 100 ng/µL of Cas9 RNA and 25 ng/µL of sgRNA no. 1. Multiple heteroduplex bands were shown in PCR amplicons from each the RGEN-injected embryo (#1–6), whereas a single band was shown from each “Control” embryo without injection of the RGENs. (D,E) Subcloned sequences observed in the RGEN-injected embryos #1 (D) and #2 (E). Red dashes and letters indicate the identified mutations. The sgRNA targeting sequence and PAM indicate in green and light blue boxes, respectively. The size of deletions and insertions are shown to the right of each mutated sequence (Δ; deletions, +; insertions). Numbers on the right edge indicate the numbers of mutated clones identified from all analyzed clones from each embryo.

To produce customized guide RNAs, we used the pDR274 vector, a T7 polymerase-mediated expression vector for a synthetic sgRNA, that was used in a zebrafish study (Hwang et al., 2013b). First, we designed an sgRNA in the second exon of the *DJ-1/park7* gene (Ensembl gene no. ENSORLG00000004285), successfully disrupted by TALENs in our previous study (Ansai et al., 2013). A sequence of the form 5′-GG-N_18_-NGG-3′ was selected for the first target (5′-GGCCTCTTCCAAGCTAGTATCGG-3′; site no. 1) according to a previously described design guideline (Hwang et al., 2013b) (Fig. 1A,B). To induce efficient expression of Cas9 nuclease, we generated a pCS2+-based Cas9 nuclease expression vector to produce a capped RNA by SP6 RNA polymerase, containing a human codon-optimized *S. pyogenes* Cas9 nuclease fused to a triple FLAG tag and two nuclear localization signal (NLS) in both N- and C-terminals previously used in cultured human cells (Cong et al., 2013; Ran et al., 2013b).

To investigate the efficiency of inducing targeted mutations in medaka, the solution containing 25 ng/µL of sgRNA and 100 ng/µL of Cas9 RNA was injected into fertilized eggs of the d-rR medaka strain. Genomic DNA was extracted from each injected embryo at 3 dpf, followed by assessment of the presence of targeted mutations by HMA. Formation of heteroduplexes was observed in all analyzed embryos (Fig. 1C), indicating that the RGEN-mediated indels were induced at the target locus. Subsequently, the *DJ-1* gene region containing the target site was PCR-amplified from two representative embryos. The PCR products were subcloned and each clone was sequenced. All the 33 sequenced clones had altered sequences, including 6 types of mutations in embryo #1-1 (17 of 17 sequenced clones; 100%) (Fig. 1D) and 7 types of mutations in embryo #1-2 (16 of 16; 100%) (Fig. 1E). These results indicate that the RGEN introduced DNA double-strand breaks at the target genomic sequence and thereby induced indels via error-prone nonhomologous end joining repair with high efficiency.

### The sgRNAs transcribed by T7 polymerase do not necessarily require the target sites starting with GG

**Fig. 2.**
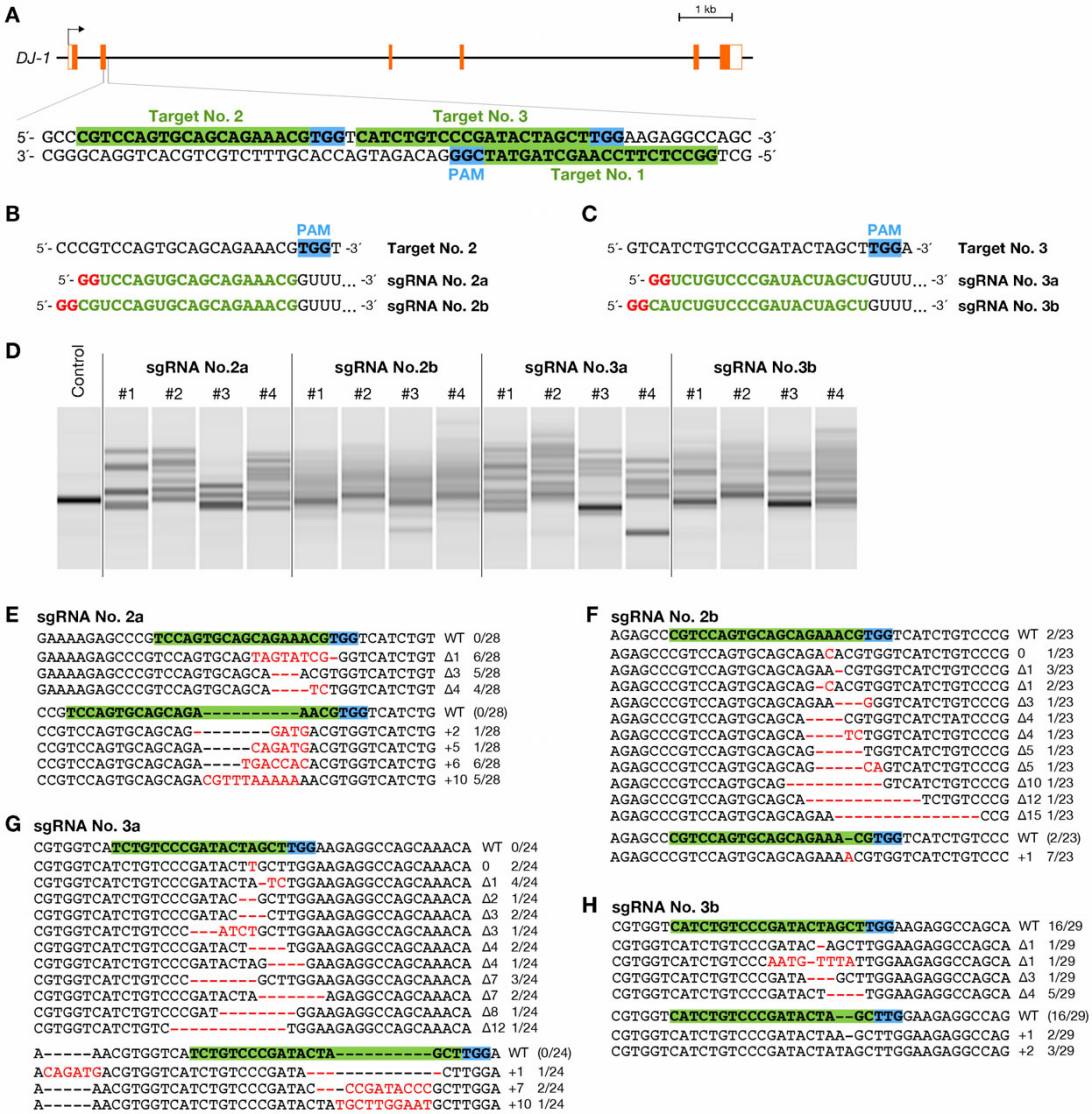
Somatic mutations induced at genomic sequences not containing GG at the 5′ ends. (A) Schematic illustration of the additional RGEN targeting sequences on the 2nd exon of *DJ-1* gene. Target site no. 2 and 3 do not contain GG at their 5′ ends, while target site no. 1 starts with the sequence GG. The targeting sequence of sgRNA is indicated in green box, adjacent to NGG protospacer adjacent motif (PAM) sequence in light blue box. (B,C) The sequences of sgRNAs for target site no. 2 (B) and 3 (C). Red and green letters indicate the sequence required by T7 RNA polymerase and the customizable targeting sequence, respectively. Two sgRNAs were designed for each target site. The sgRNA no. 2a and 3a contain the 18-nts sequence complementary to their genomic target site, while the sgRNA no. 2b and 3b contain the 20-nts sequence. These sgRNAs also contain 1- or 2-nt mismatches to their genomic target sequence at 5′ end. (D) Heteroduplex mobility assay (HMA) in embryos injected with a mixture containing 100 ng/µL of Cas9 RNA and 25 ng/µL of sgRNA. Multiple heteroduplex bands were shown in PCR amplicons from each the RGEN-injected embryo, whereas a single band was shown from each “Control” embryo without the injection of the RGENs. (E–H) Subcloned sequences observed in the embryos injected with sgRNA no. 2a (E), 2b (F), 3a (G), or 3b (H). Red dashes and letters indicate the identified mutations. The sgRNA targeting sequence and PAM indicate in green and light blue boxes, respectively. The size of deletions and insertions are shown to the right of each mutated sequence (Δ; deletions, +; insertions). Numbers on the right edge indicate the numbers of mutated clones identified from all analyzed clones from each embryo.

**Fig. 3.**
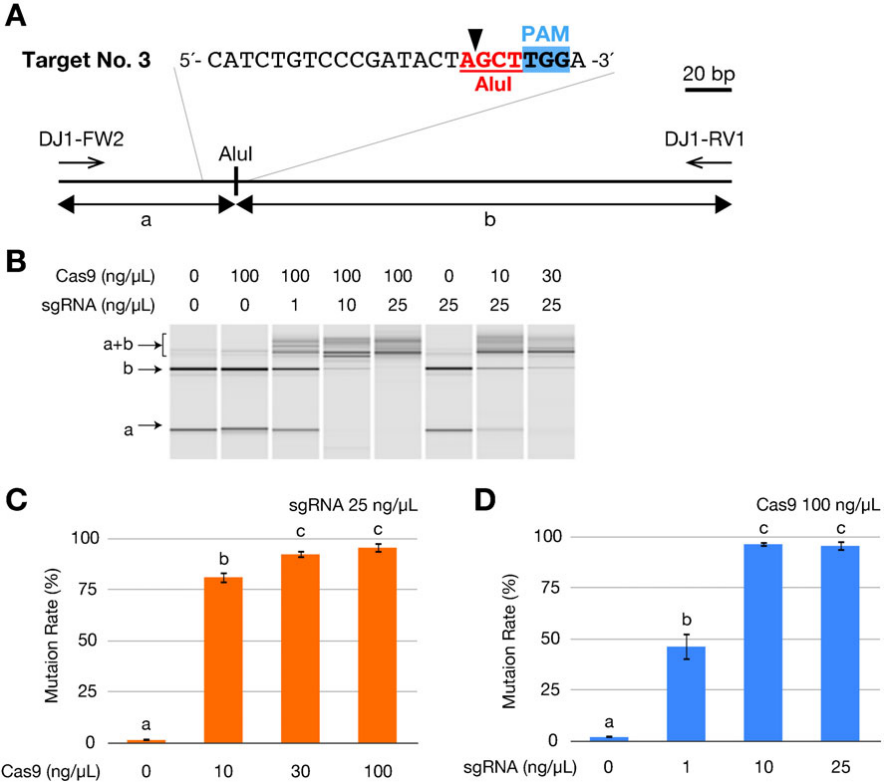
Dose-dependent mutagenesis by the RGENs. (A) Schematic illustration of restriction fragment length polymorphism (RFLP) analysis to calculate mutation frequencies. The sgRNA no. 3a contatin an AluI restriction enzyme site (Red letters with underline) on the potential cleavage site indicated by an arrowhead. A 285 bp fragment amplified using primers DJ1-FW2 and DJ1-RV1 produces both 75 bp (a) and 210 bp fragments by AluI digestion in wild type. (B) Gel image of AluI-digested fragments analyzed in MultiNA system. The RGEN-injected embryos exhibited undigested fragments (a+b). Images from a representative embryo injected with varying amounts of Cas9 RNA and sgRNA no. 3a are shown. (C,D) Mutation rates at each injected Cas9 RNA concentration with 25 ng/µL of sgRNA (C) and at each injected sgRNA concentration with 100 ng/µL of Cas9 RNA (D). The mutation rate was calculated as the molar concentration of the undigested fragment (a+b) with AluI as a percentage of the sum of molar concentrations of the undigested fragment (a+b) and the larger digested fragment (b). The molar concentration of each fragment was quantified using the MultiNA Viewer software. Columns and error bars represent mean ± s.e.m. (*n* = 12). The different letters at the top of the columns indicate significant differences (P<0.05; one-way ANOVA followed by Tukey's HSD test).

For more flexible targeting by the CRISPR/Cas system, we examined whether GG at the 5′ end of the targeting sequence is required for the sgRNAs transcribed by T7 RNA polymerase. We selected 2 genomic sequences (20 bp) followed by a PAM sequence on the second exon of the *DJ-1* gene as additional targets (Fig. 2A). Site no. 2 (5′-CGTCCAGTGCAGCAGAAACGTGG-3′) contains CG at the 5′ end and site no. 3 (5′-CATCTGTCCCGATACTAGCTTGG-3′) contains CA at the 5′ end. To design sgRNAs that target these sequences, we employed 2 strategies as follows: (a) customizing only 18 nts of the sgRNA by replacing mismatches between GG added at the 5′ end and the genomic target sequence; (b) customizing all modifiable 20-nt-long sequences of the sgRNA by ignoring the GG added at the 5′ end. The sgRNA no. 2a or 2b for target site no. 2 (Fig. 2B) and the sgRNA no. 3a or 3b for site no. 3 (Fig. 2C) were designed according to the strategy (a) or (b), respectively. We injected the solution containing 25 ng/µL sgRNA and 100 ng/µL Cas9 RNA. Results of HMA using RGEN-injected embryos showed that all 4 sgRNAs induced mutations at their targeting site (Fig. 2D). Subsequent sequence analysis using 2 representative embryos in each sgRNA not only revealed the introduction of indels, but also exhibited that the efficiency of inducing mutation depended on the design of the sgRNA (Fig. 2E–H). sgRNA no. 2a (Fig. 2E) and 3a (Fig. 2G) induced mutations with high efficiencies (28/28; 100% and 24/24; 100%, respectively), whereas sgRNA no. 2b (Fig. 2F) and 3b (Fig. 2H) induced mutations with relatively lower efficiencies (21/23; 91.3% and 13/29; 44.8%, respectively). These results exhibit that the GG at the 5′ end of the target genomic sequence is not essential for DNA cleavage by the sgRNAs transcribed by the T7 promoter. Additionally, these results indicate that the sgRNAs customized with the 18-nt sequences following the first added GG can induce targeted mutations with higher efficiency.

### Both Cas9 RNA and sgRNA induce mutations in a dose-dependent manner

To investigate the dose dependence of RGEN-induced mutation, we injected varying amounts of Cas9 RNA and sgRNA no. 3a because its targeting sequence contains an AluI restriction enzyme site that facilitates restriction fragment length polymorphism (RFLP) analysis (Fig. 3A). Most embryos injected with Cas9 RNA and/or the sgRNA developed normally and similar to the untreated embryos (Table 1). Genomic DNA was extracted from each surviving embryo at 3 dpf. Subsequently, the PCR amplicon, which included the targeted genomic sequence, was subjected to AluI digestion and analyzed by the MultiNA system. All samples that were injected with both Cas9 RNA and sgRNA showed the undigested fragment (a+b, Fig. 3B) while control samples without Cas9 RNA and/or sgRNA showed no undigested fragment but two AluI-digested fragment (a and b, Fig. 3B). Then, we calculated the disrupting activity of the RGEN at each concentration using the quantities of digested and undigested fragments. Serial dilutions of the Cas9 RNA exhibited that 10 ng/µL Cas9 RNA induced mutations with significantly lower efficiency (81.2±2.9%) as compared to 30 and 100 ng/µL (92.4±1.8% and 95.7±2.5%, respectively) (Fig. 3C). Similar results were obtained with sgRNA, as exhibited by a significantly low efficiency of mutation with 1 ng/µL sgRNA (46.4±6.6%) as compared to 10 and 25 ng/µL (96.5±1.3% and 95.7±2.5%, respectively) (Fig. 3D). These results indicate that the efficiency of both Cas9 RNA and sgRNA in inducing mutations is dose dependent.

**Table 1.**
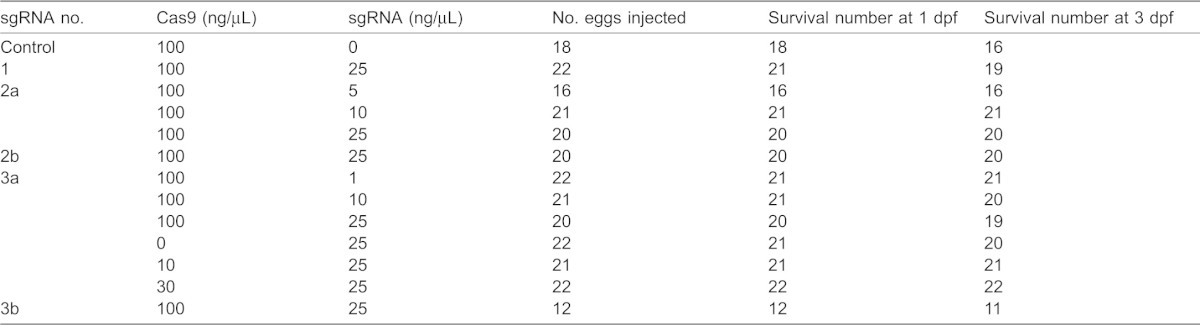
Survival of embryos injected with varying amounts of Cas9 RNA and/or sgRNA

### Off-target alterations with the RGENs in the medaka genome

**Fig. 4.**
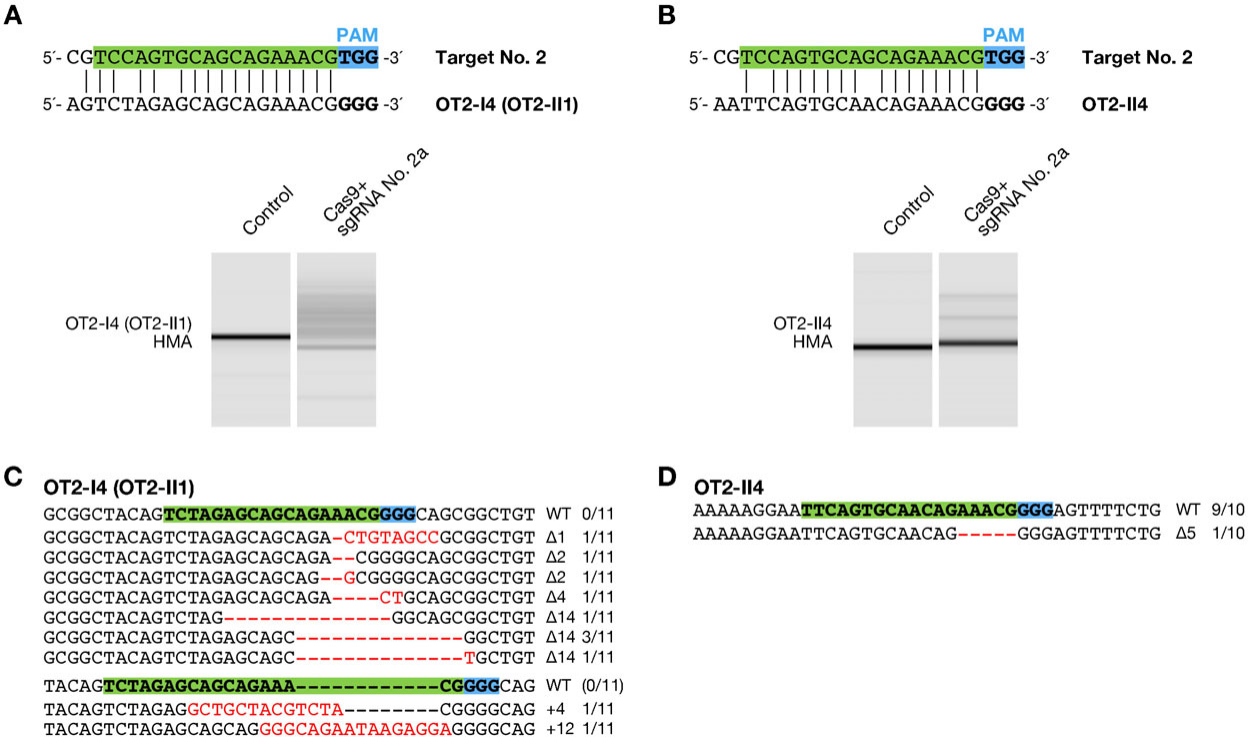
Analysis of off-target mutagenesis. (A,B) Heteroduplex mobility assay (HMA) of the mutagenized off-target loci OT2-I4 (OT2-II1) (A) and OT2-II4 (B). The upper panel shows the alignment of the off-target sites with the targeting sequence no. 2. The targeting sequence of sgRNA is indicated in green box, adjacent to NGG protospacer adjacent motif (PAM) sequence in light blue box. The lower panel shows HMA of the off-target loci using genomic DNA mixtures of the 12 embryos. Multiple heteroduplex bands were shown in PCR amplicons from embryos injected with 100 ng/µL of Cas9 RNA and 25 ng/µL of sgRNA no. 2a, whereas a single band was shown from “Control” embryos without injection of the RGENs. (C,D) Subcloned sequences of off-target alterations at OT2-I4 (OT2-II1) (C) and OT2-II4 (D) were identified in the RGEN-injected embryos. Red dashes and letters indicate the identified mutations. The sgRNA targeting sequence and PAM indicate in green and light blue boxes, respectively. The size of deletions and insertions are shown to the right of each mutated sequence (Δ; deletions, +; insertions). Numbers on the right edge indicate the numbers of mutated clones identified from all analyzed clones from each genomic DNA mixture.

To assess the possibility of induction of off-target mutagenesis by RGEN we searched candidate off-target sequences that could potentially be targeted by the 3 sgRNAs (no. 1, 2a, and 3a) for the *DJ-1* gene. Previous in vitro studies (Jinek et al., 2012) and in bacteria (Jiang et al., 2013) and human cells (Cong et al., 2013) have shown that cleavage by Cas9 can be abolished by single mismatches in the “seed” sequence, a 10–12-bp sequence located in the 3′ end of the 20-bp targeting region. We therefore searched the medaka genome for candidate sites that perfectly match the 12-bp sequence at the 3′ ends of the 20-bp targeting sequence and the NGG PAM sequence, referred to as criterion (i), (see “Off-target analysis” in Materials and Methods). We identified 4, 17, and 4 candidate sites for the sgRNA no. 1, 2a, and 3a, respectively (supplementary material Table S3). Using HMA, we detected efficient alterations in the embryos injected with the sgRNA no. 2a at a genomic locus (OT2-I4: 5′-**A**GTC**T**A**G**AGCAGCAGAAACGGGG-3′) harboring 3-bp mismatches (Fig. 4A), while no alteration was detected at other candidate sites (supplementary material Fig. S1).

Recently, Fu et al. revealed that one or more mismatches located in the 3′ half of the sgRNA targeting region are tolerated (Fu et al., 2013), and Hsu et al. also revealed that *S. pyogenes* Cas9 can cleave targets with a NAG PAM (Hsu et al., 2013). Therefore, we investigated potential off-target sites identified by another criterion. Based on our data described in the section “The sgRNAs transcribed by T7 polymerase do not necessarily require the target sites starting with GG”, which suggests that the 2-nt sequence at the 5′ end of the sgRNAs is not crucial to targeting sequence recognition of the RGENs, we searched the genome for candidate sites that match with the 18-bp sequence at the 3′ ends of the targeting sequence harboring up to 2-bp mismatches adjacent to either NGG or NAG PAM sequence, referred to as criterion (ii). We identified 4 and 8 additional candidate sites for the target site no. 1 and 2a, respectively (supplementary material Table S4), including the OT2-I4 meeting the first criterion (supplementary material Table S3), and then detected alterations in the embryos injected with the sgRNA no. 2a at a locus (OT2-II4: 5′-**AA**T**T**CAGTGCA**A**CAGAAACGGGG-3′) (Fig. 4B). DNA sequencing confirmed that mutations were induced with high efficiency at the OT2-I4 (11/11; 100%) (Fig. 4C) and low efficiency at the OT2-II4 (1/10; 10%) (Fig. 4D). These results showed that the RGENs have the potential to induce off-target mutations in vivo.

**Fig. 5.**
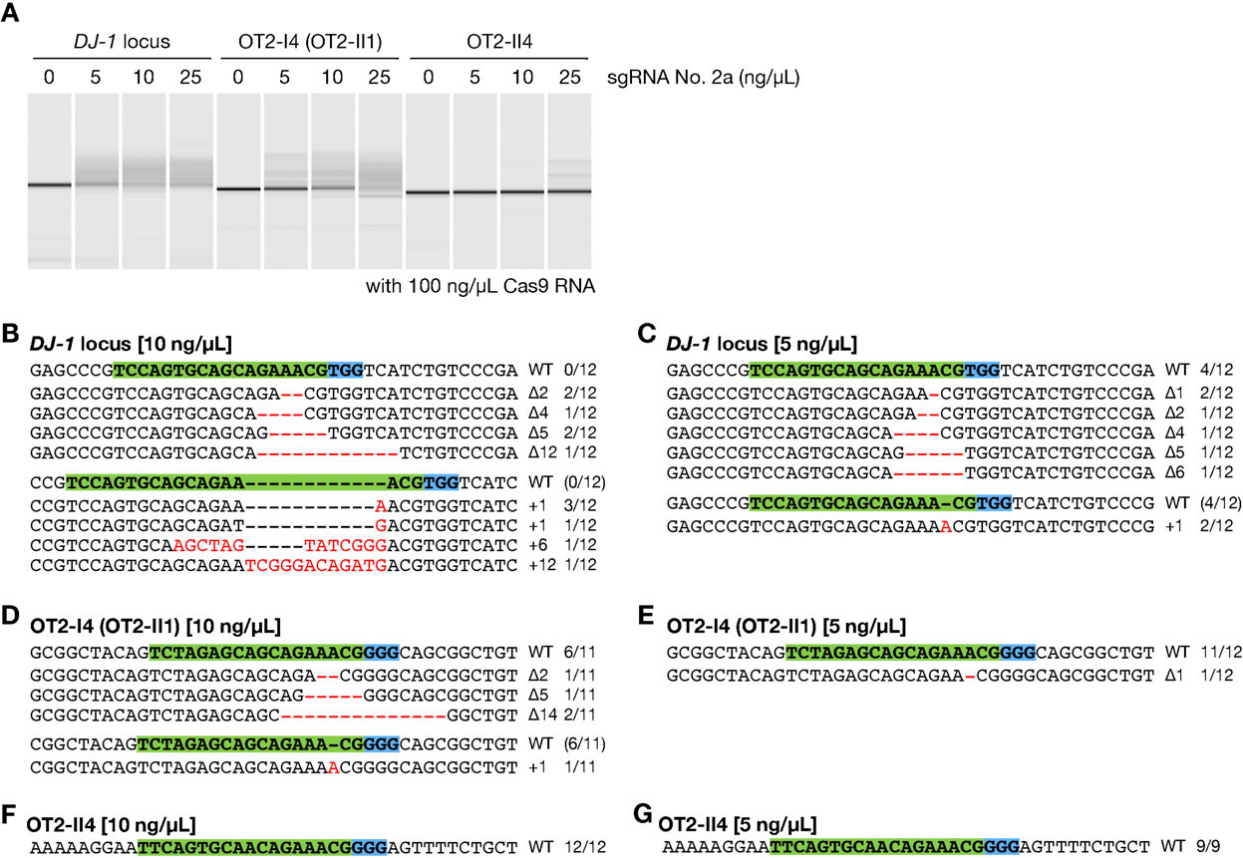
Dose-dependency of off-target alterations by the RGEN. Both the *DJ-1* targeting locus and two disrupted off-target loci were analyzed using genomic DNA mixture of the 12 embryos that were injected with dilution series of sgRNA no. 2a and 100 ng/µL Cas9 RNA. (A) Heteroduplex mobility assay (HMA). Each analyzed locus and concentration of the injected sgRNA was shown in the upper side of the panel. (B,C) Subcloned sequence of the targeting locus observed in the embryos injected with either 10 ng/µL (B) or 5 ng/µL (C) of sgRNA no. 2a. (D–G) Subcloned sequence of the off-target locus OT2-I4 (OT2-II1) (D,E) or OT2-II4 (F,G) observed in the embryos injected with either 10 ng/µL (D,F) or 5 ng/µL (E,G) of sgRNA no. 2a. (B–G) Red dashes and letters indicate the identified mutations. The sgRNA targeting sequence and PAM indicate in green and light blue boxes, respectively. The size of deletions and insertions are shown to the right of each mutated sequence (Δ; deletions, +; insertions). Numbers on the right edge indicate the numbers of mutated clones identified from all analyzed clones from each genomic DNA mixture.

Furthermore, we investigated the dose effect of sgRNA on off-target alterations using sgRNA no. 2a. Cas9 RNA (100 ng/µL) was injected with 5 or 10 ng/µL of sgRNA no. 2a and then we analyzed the *DJ-1* target locus and two off-target loci (OT2-I4 and OT2-II4), which were mutated in the previous experiment, using genomic DNA mixture from 12 embryos at 3 dpf. At the *DJ-1* locus, the lower doses of the sgRNA, 10 ng/µL (12/12; 100%) (Fig. 5B) and 5 ng/µL (8/12; 66.7%) (Fig. 5C), induced mutations as efficiently as 25 ng/µL (28/28; 100%) (Fig. 2E). On the other hand, at the off-target loci OT2-I4 and OT2-II4, the lower doses of the sgRNA, 10 ng/µL (5/11; 45.5% and 0/12; 0%, respectively) (Fig. 5D,F) and 5 ng/µL (1/12; 8.3% and 0/9; 0%, respectively) (Fig. 5E,G), dramatically reduced mutation frequencies compared to 25 ng/µL (11/11; 100% and 1/10; 10%, respectively) (Fig. 4C,D). These results showed that the sgRNA no. 2a induced mutations more efficiently at the on-target locus than the off-target loci. These also suggest that lower dosage of sgRNA is likely to reduce off-target effects by the RGENs.

### Evaluation of RGEN-induced germ line mutations

**Fig. 6.**
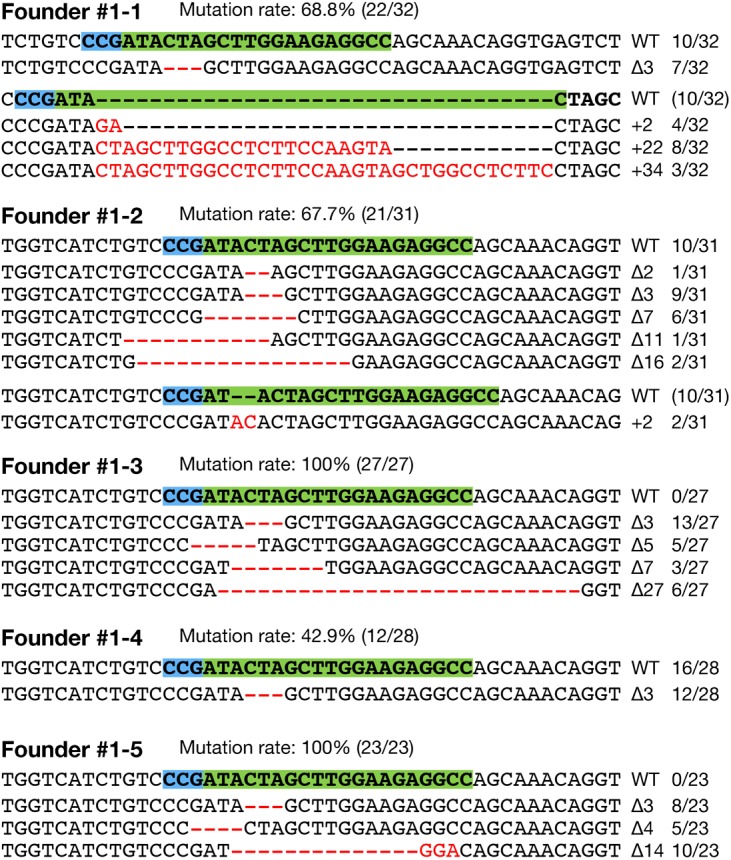
Germ line transmission of the RGEN-induced mutations. Each G0 founder that was injected with 100 ng/µL of Cas9 RNA and 25 ng/µL of sgRNA no. 1 was mated with wild-type fish to screen heritable mutations. Mutation sequences identified in each F1 embryo of them by heteroduplex mobility assay (HMA) and subsequent direct sequencing were shown. Red dashes and letters indicate the identified mutations. The sgRNA targeting sequence and protospacer adjacent motif (PAM) indicate in green and light blue boxes, respectively. The size of deletions and insertions are shown to the right of each mutated sequence (Δ; deletions, +; insertions). Numbers on the right edge indicate the numbers of mutated embryos identified from all analyzed embryos. The frequencies of mutations in each founder are indicated on the top of mutation sequences.

To test whether RGENs can induce heritable mutations, we raised the RGEN-injected fish to sexual maturity and analyzed their progeny. Of the 40 eggs injected with 100 ng/µL Cas9 RNA and 25 ng/µL sgRNA no. 1, 32 (80%) hatched normally. Five G0 fish were mated with wild-type fish of the d-rR strain, and their F1 embryos were genotyped by HMA. We identified that all 5 G0 fish transmitted the RGEN-induced mutations to their progeny. The germ line transmission rates of the mutations in each G0 fish ranged from 42.9% (12 of 28; #1–4) to 100% (27 of 27; #1–3, 23 of 23; #1–5) (Fig. 6). The mutation pattern found in the germ cells of each G0 founder varied from 1 (#1–4) to 6 (#1–2). All mutations identified in F1 embryos are shown in Fig. 6. These results indicate that the RGENs induced heritable mutations with high efficiency.

## DISCUSSION

In this study, we described a simple and efficient approach for targeted mutagenesis in medaka by using the CRISPR/Cas-mediated RGENs. The RGENs can induce targeted genomic cleavage when injected with only capped RNA encoding a Cas9 endonuclease and a sgRNA guiding the nuclease to the complementary genomic sequence. All the designed sgRNAs induced targeted somatic mutations with high efficiency (44.8–100%; average, 86.8%). G0 founders that were injected with the RGENs carried mutations in their germ cells with high efficiency. These efficiencies are as high as those in our mutagenesis study using TALENs in medaka (Ansai et al., 2013). These results mean that the RGENs function as an efficient engineered nuclease system in medaka.

The previously designed guidelines for an sgRNA transcribed by T7 RNA polymerase requires any sequence of the form 5′-GG-N_18_-NGG-3′ that occurs once in every 128 bp of a random DNA sequence (Hwang et al., 2013b). Efficient induction of mutations by the sgRNAs designed for the target site no. 2 and 3 has revealed that mismatches between the 2-nt sequence at the 5′ ends of the sgRNAs and the target genomic sequence are tolerated. It was also reported that double mismatches at the 5′ ends are tolerated in zebrafish (Hwang et al., 2013a), suggesting that the RGEN can target any sequence only adjacent to a NGG PAM sequence that occurs once in every 8 bp. In this study, the sgRNAs starting with the sequence 5′-GG-N_18_-3′ (no. 2a and 3a) induced mutations more efficiently as compared to the sequence 5′-GG-N_20_-3′ (no. 2b and 3b). From the results, we propose the guideline for design of sgRNAs that can efficiently disrupt target genomic sequences in medaka (Table 2). On the other hand, one of the 3 examined sgRNA with the sequence 5′-GG-N_20_-3′ more efficiently induced mutations as compared to the sequence 5′-GG-N_18_-3′ in the zebrafish study (Hwang et al., 2013a). Since the effects of the 5′ ends of sgRNAs on cleaving activities were investigated in only a few examples, more comprehensive investigations will be required to generalize the effectiveness of the 5′ structures to other targeting sites.

**Table 2.**
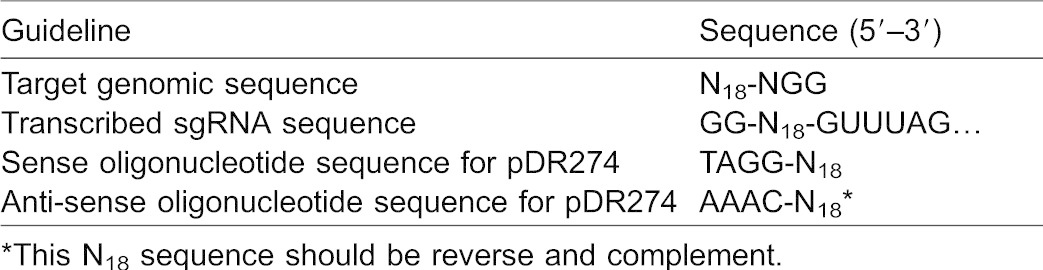
A proposed guideline for design of efficient sgRNAs transcribed by the T7 promoter

Previous in vivo works defined a 12-bp sequence adjacent to a PAM as a seed sequence and counted the genomic sequences that perfectly matched the seed sequence as potential off-target sites (Bassett et al., 2013; Friedland et al., 2013; Gratz et al., 2013; Wang et al., 2013). However, the study in human cells revealed that one or more mismatches located in the 12-bp sequences are tolerated (Cho et al., 2014; Fu et al., 2013; Wang et al., 2014), suggesting that some in vivo off-target alterations are likely to be missed in these works. In fact, although one of the 2 mutagenized off-target loci (OT2-I4, Fig. 4) meets this criterion (called “criterion (i)” in this study), the other locus (OT2-II4, Fig. 4), harboring a single mismatch in the 12-bp sequence of a seed sequence, does not meet this criterion. Both these mutagenized off-target loci meet the criterion (ii), matching 16 to 18 of 18-bp sequence at the 3′ end of the targeting sequence followed by a NRG (either the NGG or NAG) PAM.

It was reported that potential off-targets meeting the criterion (ii) were efficiently disrupted in cultured human cells (Cho et al., 2014; Fu et al., 2013; Hsu et al., 2013; Wang et al., 2014) and zebrafish (Jao et al., 2013). Altogether, we propose that potential off-target loci identified by the criterion (ii), harboring 2- or fewer bp mismatches in the 18-bp targeting sequence followed by a NRG PAM, should be analyzed for screening unwanted mutagenesis. However, Cho et al. showed that some RGENs can distinguish on-target sites from off-target sites that differ by at least two bases (Cho et al., 2014). Both this report and our limited analysis suggest that further investigations (e.g. genome-wide or deep-sequencing analysis) will be required to determine the generalized criteria for searching the off-target sites in medaka and other organisms.

Significant induction of in vivo off-target alterations indicates that the potentially confounding effects of off-target mutations should be considered in analysis using the RGEN-mediated genome-edited organisms. Also, since our approach for searching of potential off-target sites is based on similarities to the targeting sequences, we may miss some off-target effects in this study. However, in this study, fish injected with the RGENs developed normally (Table 1), suggesting that their off-target effects are not crucial for viability and have little non-specific toxicity unlike found in the fish injected with ZFNs (Ansai et al., 2012). Additionally, each sgRNA that was designed in this study has unique profile of off-target activities and only the 2 loci for sgRNA no. 2 were mutagenized (Fig. 4; supplementary material Fig. S1). These suggest that validating the phenotypes with at least two independent lines generated by different sgRNAs is important to eliminate the off-target effects, in addition to cleaning the background mutations by repeated outcrossing and rescue experiments. Furthermore, we found that 5 or 10 ng/µL of sgRNA no. 2a induced off-target alterations with significantly lower efficiencies than did 25 ng/µL of the sgRNA (Fig. 5). This indicates that injection with lower dosage of sgRNAs will also become an effective way to reduce the off-target effects.

At present, ZFNs, TALENs, and CRISPR/Cas-mediated RGENs are 3 successful options for targeted genome editing in medaka (Ansai et al., 2012; Ansai et al., 2013; Chen et al., 2012). Both the TALEN and the CRISPR/Cas systems allow for the construction of highly efficient nucleases that target desired sequences more easily than the ZFN system. Compared to the TALENs, the CRISPR/Cas system has some advantageous characteristics for efficient genome editing. Firstly, determination of DNA cleavage specificity by guide RNA sequences allows the customizing of the RGENs only by modification of the 5′ end of the sgRNA using annealed oligonucleotides, while the customizing of TALENs requires preparation of multiple vectors (at least 35 vectors, as described earlier (Ansai et al., 2013; Sakuma et al., 2013)) and complex assembly processes. Secondly, the RGENs can efficiently cleave DNA targets containing CpG methylation sites (Hsu et al., 2013), while TALENs are sensitive to methylation (Valton et al., 2012). Finally, Cas9 nickases can be simply engineered by the introduction of a point mutation into Cas9 nuclease (e.g. D10A) (Jinek et al., 2012), which are likely to efficiently induce precise genome engineering via homology-directed repair without the effects of indel mutations induced via non-homologous end joining pathway (Cong et al., 2013). These indicate that the CRISPR/Cas-mediated RGENs have the potential to be developed as an alternative technology for targeted genome editing in medaka and other organisms.

## Supplementary Material

Supplementary Material
