# Supplementary material for: Targeted mutagenesis using CRISPR/Cas system in medaka
Source: Biol Open. 2014 Apr 11;3(5):362–71. doi: 10.1242/bio.20148177 (PMC4021358; doi:10.1242/bio.20148177)
Supplement: Supplementary Material [file supp_3_5_362__index.html]

Targeted mutagenesis using CRISPR/Cas system in medaka — Supplementary Material 

# Targeted mutagenesis using CRISPR/Cas system in medaka

## bio.20148177 Supplementary Material

**Files in this Data Supplement:**

- Supplementary Material - Satoshi Ansai and Masato Kinoshita doi: 10.1242/bio.20148177
