# Supplementary material for: Targeted mutagenesis using CRISPR/Cas system in medaka
Source: Biol Open. 2014 Apr 11;3(5):362–71. doi: 10.1242/bio.20148177 (PMC4021358; doi:10.1242/bio.20148177)
Supplement: Supplementary Material [file supp_bio.20148177_bio.20148177-s1.pdf]

Biology Open (2014) 000, 1–10 doi:10.1242/bio.20148177

Supplementary Material

Satoshi Ansai and Masato Kinoshita doi: 10.1242/bio.20148177

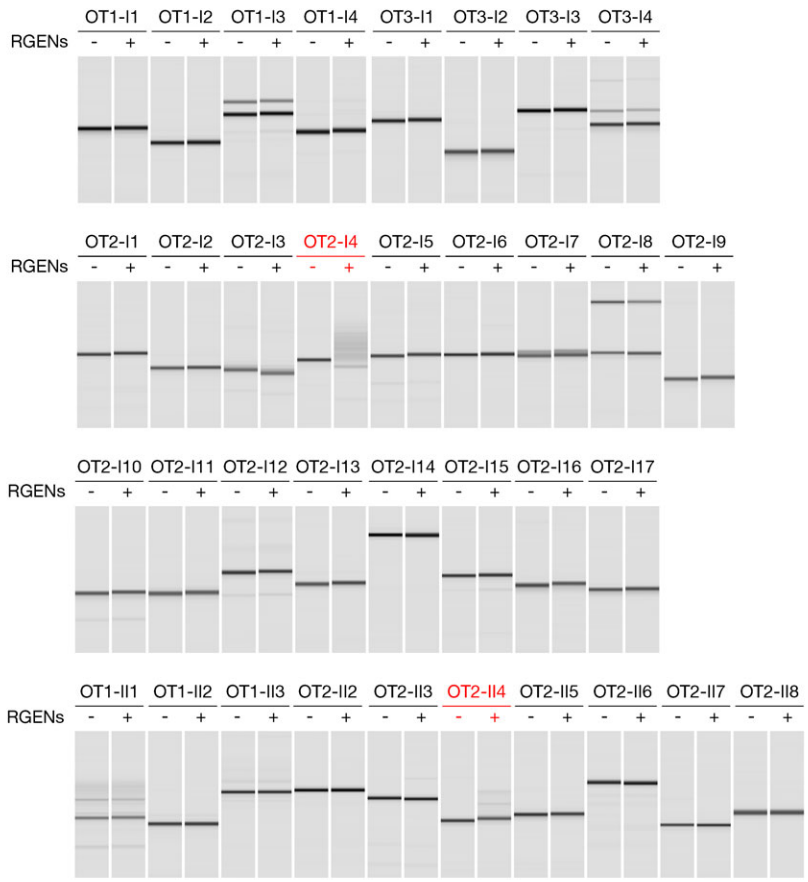

Fig. S1. MultiNA gel images of heteroduplex mobility assay (HMA) for detecting off-target alterations. Potential off-target loci for target no. 1 (OT1), no. 2 (OT2), or no. 3 (OT3) were analyzed using the mixture of genomic DNA extracted from 12 embryos that were injected with 100 ng/µL of Cas9 RNA and either 25 ng/µL of sgRNA no. 1, 2a, or 3a, respectively. I and II indicate the potential off-target loci identified by criteria (i) and (ii). Sequences of the off-target loci and primers used for HMA were listed in supplementary material Tables S3 and S4. Red letters indicate two mutagenized off-target loci (OT2-I4 and OT2-II4).

Table S1. Primer sequences used in this study

Name	Sequence (5′–3′)	Usage
hSpCas9FW	GCAGGATCCGCCACCATGGACTATAAGGAC	Cloning of hSpCas9 into the pCS2+ vector
hSpCas9RV	AGTTCTAGATTACTTTTTCTTTTTTGCCTGGC	Cloning of hSpCas9 into the pCS2+ vector
DJ1-FW2	TGTGACTGTAGCGGGTCTGA	HMA, RFLP
DJ1-RV1	GTGTGAACAACGCTGCATTT	RFLP, direct sequencing
DJ1-RV2	GTCGCAATTGCCATCACTTT	HMA
DJ1-FW3	CAGGAATTCTTTTTGTCTCTTGGTGGTGCT	Subcloning into the pBluescript vector
DJ1-RV3	CCCCTCGAGGTGTGAACAACGCTGCATTT	Subcloning into the pBluescript vector
M13-FW	GTAAAACGACGGCCAGT	Sequencing
M13-RV	GGAAACAGCTATGACCATG	Sequencing
T7 promoter	TAATACGACTCACTATAGGG	Sequencing
SP6 promoter	TATTTAGGTGACACTATAG	Sequencing

Table S2. Oligonucleotide sequences used for construction of sgRNAs

sgRNA ID	Direction	Sequence (5′–3′)
No. 1	Sense	TAGGCCTCTTCCAAGCTAGTAT
	Anti-sense	AAACATACTAGCTTGGAAGAGG
No. 2a	Sense	TAGGTCCAGTGCAGCAGAAACG
	Anti-sense	AAACCGTTTCTGCTGCACTGGA
No. 2b	Sense	TAGGCGTCCAGTGCAGCAGAAACG
	Anti-sense	AAACCGTTTCTGCTGCACTGGACG
No. 3a	Sense	TAGGTCTGTCCCGATACTAGCT
	Anti-sense	AAACAGCTAGTATCGGGACAGA
No. 3b	Sense	TAGGCATCTGTCCCGATACTAGCT
	Anti-sense	AAACAGCTAGTATCGGGACAGATG

Table S3. Potential off-target sequences identified by criterion (i) in medaka genome

Target	Site name	Sequence (5′–3′)	Chromosome position	Forward PCR primer	Reverse PCR primer
sgRNA no. 1	Target 1	GGCCTCTTCCAAGCTAGTATCGG	chr5: 7,477,613-7,477,591	TGTGACTGTAGCGGGTCTGA	GTCGCAATTGCCATCACTTT
	OT1-I1a	CGTGTAACCCAAGCTAGTATTGG	chr8: 4,153,407-4,153,385	AAAGTTTTTCTGGTCCCATGT	GTAAGGGTAAAGGGGCTGCT
	OT1-I1b	CGTGTAACCCAAGCTAGTATTGG	chr8: 4,156,178-4,156,156	AAAGTTTTTCTGGTCCCATGT	GTAAGGGTAAAGGGGCTGCT
	OT1-I2	TAGGTACACCAAGCTAGTATCGG	chr8: 9,508,743-9,508,765	TGCCACCAAGGATTAAGACA	CCCCTGCATGATTTCAGTTT
	OT1-I3	CTATATTACCAAGCTAGTATTGG	chr9: 15,422,034-15,422,012	CTGGCTTTTCAAGGCGAATA	GGCCATCTTCTTCAGTCTGG
	OT1-I4	GTTTTGCACCAAGCTAGTATCGG	chr16: 21,913,658-21,913,680	AGAAAAAGCCTGGAGCAAAA	TACGCTCAGACCGGGTACA
sgRNA no. 2a	Target 2	CGTCCAGTGCAGCAGAAACGTGG	chr5: 7,477,559-7,477,581	TGTGACTGTAGCGGGTCTGA	GTCGCAATTGCCATCACTTT
	OT2-I1	CTCCTCCGGCAGCAGAAACGGGG	chr1: 34,070,805-34,070,827	GGCAATCAAGGAACAGAATGA	TAAAGTCCCCTCCTCCCTTC
	OT2-I2	AGAGCATCGCAGCAGAAACGAGG	chr1: 38,622,323-38,622,301	CCGCAAACTCCAGTCAGTCT	CGTGAAATTCCCCAGAAGTC
	OT2-I3	CTGAAGCTGCAGCAGAAACGGGG	chr4: 6,616,842-6,616,820	GGACTGGACAGTTTGGAGGA	TGATGTGAAGGTGGACATGC
	OT2-I4	AGTCTAGAGCAGCAGAAACGGGG	chr4: 20,089,705-20,089,727	AGAAAGTCGGTGGTAACAGCA	TCTAGACTGCGGCATGTGAC
	OT2-I5	TAGGAGCAGCAGCAGAAACGAGG	chr5: 24,232,491-24,232,469	AGAGCAGAGACGAGCCAGAG	AGGGGATCAACAACAATGGA
	OT2-I6	CAAGGACAGCAGCAGAAACGCGG	chr8: 14,070,959-14,070,981	AGGCAACAGGAGGAGAAACA	GCTGCTGGAGGAGGTATCTG
	OT2-I7	CAAATGTTGCAGCAGAAACGCGG	chr8: 17,753,755-17,753,733	GCAGGGTGATACAGGATGGA	TCACCGATTTACCAAATTTGAAC
	OT2-I8	TGCAGATTGCAGCAGAAACGTGG	chr9: 29,205,714-29,205,692	GGACATTTTTAAACACCCCAAA	GGGACATGTGCGCATATAAA
	OT2-I9	AGCATGACGCAGCAGAAACGAGG	chr9: 30,256,570-30,256,592	AAGGCCGATAACAAGACTTCC	CCTGTGTGAGGAAGATGAAGG
	OT2-I10	CACTCCCTGCAGCAGAAACGCGG	chr10: 26,433,826-26,433,804	GGGATTGGAGGAGGTTCATC	CAGGCTGAACATCATTTTGG
	OT2-I11	CATGAGGTGCAGCAGAAACGGGG	chr12: 2,746,166-2,746,144	TGTCATGTTCCTTTTTGTGTCC	CAACACGACCTGAAATGTGTG
	OT2-I12	TGATTTGCGCAGCAGAAACGTGG	chr13: 2,841,517-2,841,539	GCGCAATTCACATCCAAAAT	TGTCTTTAATGTGCAGCCAAA
	OT2-I13	CTGGAGCCGCAGCAGAAACGTGG	chr13: 19,092,675-19,092,653	GTTCTCTCCTCATGGCGTTG	GCCGAGTTCCTTTGACTGAC
	OT2-I14	ATGAAAAAGCAGCAGAAACGGGG	chr15: 14,490,114-14,490,136	GCTGAAATGTAATTTGGAGCA	CAGCCAATGTCTAAATGCTGTC
	OT2-I15	CTGGAAATGCAGCAGAAACGGGG	chr18: 29,247,728-29,247,706	GGACGGTAAACTTCGTCTGG	TTCTGGGAGTTTGAGTGAAGC
	OT2-I16	TTCTGAGAGCAGCAGAAACGGGG	chr23: 3,690,537-3,690,559	GTTCTGTCATTCCCCTGAGC	CAAGAAACCTGCAGGAGGAG
	OT2-I17	ATTTCTTGGCAGCAGAAACGGGG	chr24: 12,802,667-12,802,645	AAGAAATGCTGGGGATGTTG	GCAAACACGTTTCCCAAACT
sgRNA no. 3a	Target 3	CATCTGTCCCGATACTAGCTTGG	chr5: 7,477,583-7,477,605	TGTGACTGTAGCGGGTCTGA	GTCGCAATTGCCATCACTTT
	OT3-I1	GCTCCAACCCCGATACTAGCTTGG	chr8: 9,508,773-9,508,751	CCCCTGCATGATTTCAGTTT	TGCCACCAAGGATTAAGACA
	OT3-I2	GTAGCTGCCCGATACTAGCTAGG	chr10: 8,557,430-8,557,408	CACATGTGTTCAAGGTGTTCA	TGTGACACGATCTGAACCTGA
	OT3-I3	GACTCTACCCGATACTAGCTTGG	chr16: 21,913,688-21,913,666	TACGCTCAGACCGGGTACA	AGAAAAAGCCTGGAGCAAAA
	OT3-I4	TGACCTATCCGATACTAGCTAGG	chr18: 1,045,229-1,045,207	GCCTTCAGCCCTCTGATGA	CCTTTGGATCCATCAACGTC

Primer sequences for mutation analysis are also shown. Red letters indicate a mutagenized off-target locus.

Biology Open (2014) 000, 1–10 doi:10.1242/bio.20148177

Biology Open (2014) 000, 1–10 doi:10.1242/bio.20148177

Table S4. Potential off-target sequences identified by criterion (ii) in medaka genome

Target	Site name	Sequence (5′–3′)	Chromosome position	Forward PCR primer	Reverse PCR primer	
sgRNA no. 1	Target 1	GGCCTCTTCCAAGCTAGTATCGG	chr5: 7,477,613-7,477,591	TGTGACTGTAGCGGGTCTGA	GTCGCAATTGCCATCACTTT	
	OT1-II1	**TTT**CTC**A**TCCAAGCTAGTATCAG	chr7: 8,868,188-8,868,210	CCTCCCCTGCAGACAAATAG	CTAGCTCACAGCCCCTCTCA	
	OT1-II2	**TT**CCTC**G**TCCAAGCT**G**GTATCAG	chr16: 8,282,950-8,282,932	TGCCACCAAGGATTAAGACA	CCCCTGCATGATTTCAGTTT	
	OT1-II3	**TT**CCTC**A**TCCAAGCTAG**C**ATCAG	chr23: 5,103,587-5,103,609	CTGGCTTTTCAAGGCGAATA	GGCCATCTTCTTCAGTCTGG	
	OT1-II4*	**TT**CCTC**A**TCCAAGCTAG**C**ATCAG	chr15: 20,255,083-20,255,063	TGCTCTGCTCCATTCTGATG	CACTAGCTTACAGCCCCTCAA	No PCR amplicon was obtained with this pair of primers.
				ACAAGTTCCCTGCTCTGCTC	TTTGCACTCTCTCCCACTAGC	No PCR amplicon was obtained with this pair of primers.
sgRNA no. 2a	Target 2	CGTCCAGTGCAGCAGAAACGTGG	chr5: 7,477,559-7,477,581	TGTGACTGTAGCGGGTCTGA	GTCGCAATTGCCATCACTTT	
	OT2-II1 (OT2-I4)	**A**GTC**T**AG**A**GCAGCAGAAACGGGG	chr4: 20,089,705-20,089,727	AGAAAGTCGGTGGTAACAGCA	TCTAGACTGCGGCATGTGAC	
	OT2-II2	**GC**TCCAGTGCAGCAGA**CAT**GTAG	chr5: 18,780,520-18,780,498	GATGATGTGCCAGAGGAAAAA	TCCCTGCTACAGGTCACCAT	
	OT2-II3	**AAA**CCAGTGC**T**GCAGAAACGTGG	chr9: 10,268,750-10,268,728	AGAGTGAAGCGTCACACCTG	AAAGTTTGCATGGGGTTCTG	
	OT2-II4	**AA**T**T**CAGTGCA**A**CAGAAACGGGG	chr12: 11,210,109-11,210,131	GAAGGTAGTGGCCACCAGAG	TTGCTGTGGAGACGTGGTTA	
	OT2-II5	**GC**TCCAG**G**GCA**A**CAGAAACGAAG	chr13: 25,743,533-25,743,555	ACCTAGCAGATCGGCTTTCA	GGTTCAAAGTCTCCCCCTCA	
	OT2-II6	C**T**TCCA**T**TGCAGCAGAAA**T**GCAG	chr15: 17,930,301-17,930,279	AGTGGATTGCAACCAATGAA	TGGAGACACATCAAATGTATTACTCA	
	OT2-II7	**AC**TCCAGTGCA**T**CAGAAA**G**GAGG	chr15: 23,412,400-23,412,422	CGGTAGCTCCTTCCACAAAC	AACAAGCCATCTGCAGTCCT	
	OT2-II8	**TT**TCCAGTGCAGCAG**G**AA**G**GCAG	chr18: 1,639,052-1,639,030	AGAGTGTTAAACCCCGACGA	GGTGACAATCCTGTTATCCACA	

Primer sequences for mutation analysis are also shown. Red letters indicate mutagenized off-target loci.
*This off-target site could not be analyzed by heteroduplex mobility assay.
